# Design of a trichogramma balls UAV delivery system and quality analysis of delivery operation

**DOI:** 10.3389/fpls.2023.1247169

**Published:** 2023-12-05

**Authors:** Hang Xing, Mengjie Li, Yijuan Qin, Gangao Fan, Yinwei Zhao, Jia Lv, Jiyu Li

**Affiliations:** College of Engineering, South China Agricultural University, Guangzhou, China

**Keywords:** UAV, trichogramma ball, delivery system, operational quality, biological control

## Abstract

The field boundaries in our country are complex. In attempts to control pests via trichogramma-dominated biological control, the long-term practice of manual trichogramma release has resulted in low control efficiency, thereby impeding sustainable agricultural development. Currently, the novel approach involves utilizing Unmanned Aerial Vehicles (UAVs) for trichogramma balls delivery; however, the system is still in its nascent stages, presenting opportunities for enhancement in terms of stability and accuracy. Furthermore, there is a notable absence of comprehensive operational quality assessment standards. In this study, we establish a stable and accurate trichogramma balls delivery system using a four-axis plant protection UAV and introduce a comprehensive evaluation method for trichogramma balls delivery system. When dealing with fields with complex boundaries, it is beneficial to divide them into rectangular, trapezoidal, and stepped small fields at the boundary and perform operations within these small fields. According to our proposed evaluation method, when only considering the effect of field operations, the most effective boundary division shape is trapezoidal, followed by rectangular. and the worst is stepped. If both field operation effectiveness and the utilization effect of placed trichogramma balls are considered, the optimal shape is trapezoidal, then stepped, with rectangular being the least effective. Consequently, for UAV sub-area operations in complex boundary fields, it is advisable to divide the boundaries into trapezoids wherever possible. Field experiment results indicate that the system’s delivery area can reach up to 4158 m²/min and the coverage rate of released trichogramma balls can exceed 97%. The system design methodology and comprehensive operational quality evaluation method proposed in this article provide technical support and scientific basis for the application and promotion of UAV delivery trichogramma balls system. This is conducive to the high-quality development of agriculture.

## 1 Introduction

In global agricultural production, agricultural pests are one of the key factors leading to food loss, causing significant economic losses to the development of agriculture. The long-term use of chemical pesticides in the current pest control process has led to resistance in field populations to commonly used pesticides; insecticides also pose problems such as killing natural enemies, causing pesticide residues and water pollution, which restrict the sustainable development of agriculture (Cheng and Zhu, 2017). Therefore, in order to ensure the safety of agricultural production and achieve sustainable development in the long run, the methods of pest control need to be transformed.

With the enhancement of people’s awareness of environmental protection and the continuous development of green agriculture, biological control methods are replacing traditional pesticide control and have become an important means for the green prevention and control of crop pests (Gauthier et al., 2019; Kumar and Arthurs, 2021). Biological control is mainly to put natural enemies of crop pests in the field, so that a large number of them are scattered in the field, so as to use parasitic, predation and other methods to reduce the number of pests, thereby reducing food loss and improving crop quality (Wang et al., 2019). This method is environmentally friendly and pollution-free while protecting agricultural products. Trichogramma species make up one of the most commonly used groups of natural enemies for biological control programs worldwide(Babendreier et al., 2019; Zang et al., 2021), they have a wide range of hosts, a wide distribution range, and a fast reproduction rate, then play an important role in disease and pest control of rice, corn, sugarcane, vegetables and some trees (Xu et al., 2016). At present, the main methods of releasing trichogramma include manual spraying trichogramma suspensions, manual hanging trichogramma cards, and manual delivering trichogramma releasers, etc. It is difficult to achieve the expected goals in terms of uniformity, coverage, and delivery speed using this long-term manual delivery method. At the same time, there is a significant lag in the strong timeliness of biological control, and the high labor cost also hinders the widespread use of this method.

In recent years, with the continuous development of agricultural machinery, mechanized equipment that can be used to deliver trichogramma is gradually emerging, mainly including ground equipment and aviation equipment.

The ground equipment mainly adopts air blowing to drop carriers carrying trichogramma or to spray trichogramma suspensions. Giles et al. designed an air blown delivery device installed on tractors after multiple improvements, the device included a storage container, a rotating metering plate, and an air-cleared ejection port. As the metering plate rotated, the formulation filled cylindrical cells in the plate. As the filled cells passed an opening on a stationary bottom plate, the cell contents fell downward while a brief pulse of compressed air insured that each cell was cleared. Release rate of the mites was controlled through rotational speed of the metering plate and the size of cells on the plate. In laboratory and field tests, this method will not disrupt the activity of natural enemies. The implementation of this method has significantly enhanced operational efficiency (Giles et al., 1995). Dionne et al. designed and tested a boom sprayer to spray trichogramma ostriniae in sweet corn canopy under real field conditions. The sprayer was equipped with a 380L water tank, and 8 XR11008-VP nozzles were installed on a 6.71m spray rod. A diaphragm pump with a flow rate of 18.5L/min was used for pressure supply. During operation, the equipment was mounted on tractors or other implements. Field trial results showed that spraying was 1.7 times faster than the manual introduction of Trichocards, and the results indicated that spraying is a promising technique for an efficient and viable introduction of parasitized eggs (Dionne et al., 2018). The above research has proven that the efficiency of using ground equipment to release Trichogramma is higher. However, the control mechanism of trichogramma determines that it needs to be placed multiple times during the crop growth cycle. The ground machine entering the field multiple times can easily compact the soil, and it has poor trafficability in fields with tall crops, making it unsuitable for operations in late stages of crop growth such as corn. As a result, some researchers began to investigate the use of aviation equipment to delivery trichogramma. Aviation equipment mainly includes manned aircraft and UAVs.

In terms of using manned aircraft, An automatic insect release system (AIRS) was developed by the International Institute of Tropical Agriculture in collaboration with Firma Dieringer of Austria and Ciba-Pilatus (now Zimex Aviation) of Switzerland for the aerial distribution of natural enemies of two cassava pests. The AIRS, installed in a twin turbo-prop aircraft, had undergone a series of tests to evaluate suitable packaging substrates, pre- and post-release mortality, biological performance and ground distribution of natural enemies (Herren et al., 1987). Bzowska-Bakalarz et al. attempted to use the Aviation Artur Trendak Zen-1 ultra light rotorcraft to control corn borers in farmland. They installed trichogramma dispensers at both ends of the fuselage rack, with a single operational area up to 80hm^2^ and a flight speed up to 100km/h, and several years of field application have shown that the prevention effect against corn borers has reached 60% to 85% (Bzowska-Bakalarz et al., 2020). The above method of using manned aircraft for operation is not only efficient, but also does not contact with crops and the ground, so can be used as a good supplement to the ground delivery machinery. However, it has high cost, high flight speed and altitude, large operating width, large turning radius, and is not suitable for complex small plot operations.

Unmanned aerial vehicles can effectively solve the problem of poor trafficability in tall stalk crop fields and soil compaction by traditional ground machinery. Compared to manned aircraft, UAVs have the advantages of small size, easy use, no need for special take-off runways, low cost, and greater promotion potential, which makes them promising for a wide range of applications (Parra, 2014; Yang et al., 2019; Chen et al., 2020; Iost et al., 2020). Li et al. applied drone technology to the delivery of trichogramma, and sprayed the egg suspension of trichogramma by peristaltic pump under pressure through the UAV spraying system. The test proved that spraying trichogramma eggs with plant protection drones had no significant effect on its activity (Li et al., 2013). Xu et al. conducted a release test of trichogramma using a six-axis multirotor UAV. Their test results showed that the coverage rate of the trichogramma deployed reached 100%, with an average hatching rate of 75.81%. However, there was a lack of analysis on the actual field coverage rate and the overall quality of field operations (Xu et al., 2016). Martel et al. used Entobot biological delivery aircraft to drop trichogramma in the forest, the experimental results showed that compared with manual delivery, UAV delivery cost was higher, but delivery was rapid and a lot of time was saved, and the control effect of the two methods was almost the same (Martel et al., 2021). Zhan et al. carried out the field delivery test based on the M45 UAV equipped with special ground station, sky station and mobile phone client, they realized the functions of path planning, recording the coordinates of the delivery point and generating operational map. The actual coverage of the test site reaches 99.33%, which can meet the operational requirements (Zhan et al., 2021). Although a lot of research has already been done, in order to further improve the field operational quality of UAVs for biological control, the stability and delivery accuracy of UAVs carrying and releasing insect systems need to be improved, and the comprehensive operational quality evaluation method needs to be clarified.

**Figure 1:**
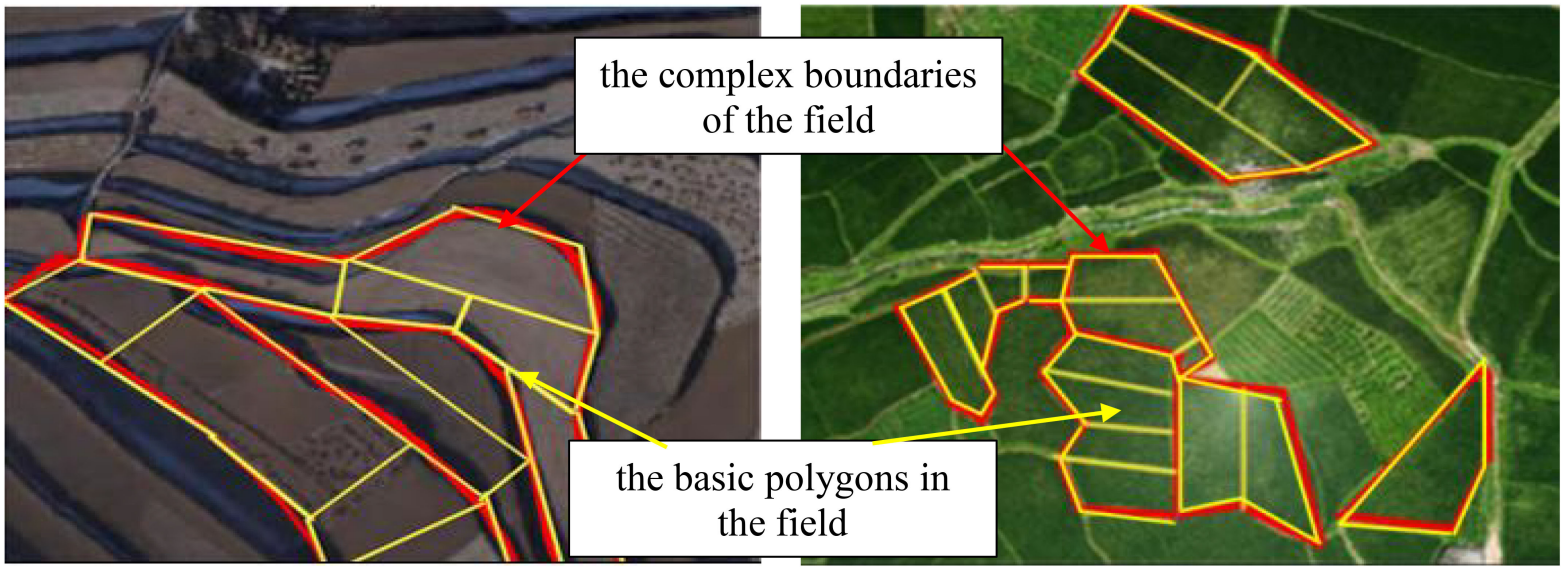
Map of natural fields with complex boundaries.

At the same time, the shape of natural farmland in China is affected by climate, terrain, water source and other factors, and most of them exhibit complex boundaries. As shown in **Figure 1**, the complex boundaries of the field are marked with red lines, and the basic polygons in the field are marked with yellow lines. During the operation of UAVs, the entire field will be divided into several small fields for operation. So the operational quality and efficiency in each small field will become the main factors affecting the comprehensive operational quality of UAVs.

In this article, we designed a new trichogramma balls delivery system based on a four-axis multirotor UAV, independently designed its delivery device and control system, completed the stability and accuracy analysis of the system and its operational efficiency tests in various field plots, especially proposed a comprehensive operational quality evaluation system to evaluate the results of field trials of the system, and pointed out which shape should be divided at the boundary when dividing complex boundary fields from the perspective of improving the comprehensive operational quality.

## 2 Materials and methods

### 2.1 Design of the trichogramma balls delivery system

**Figure 2:**
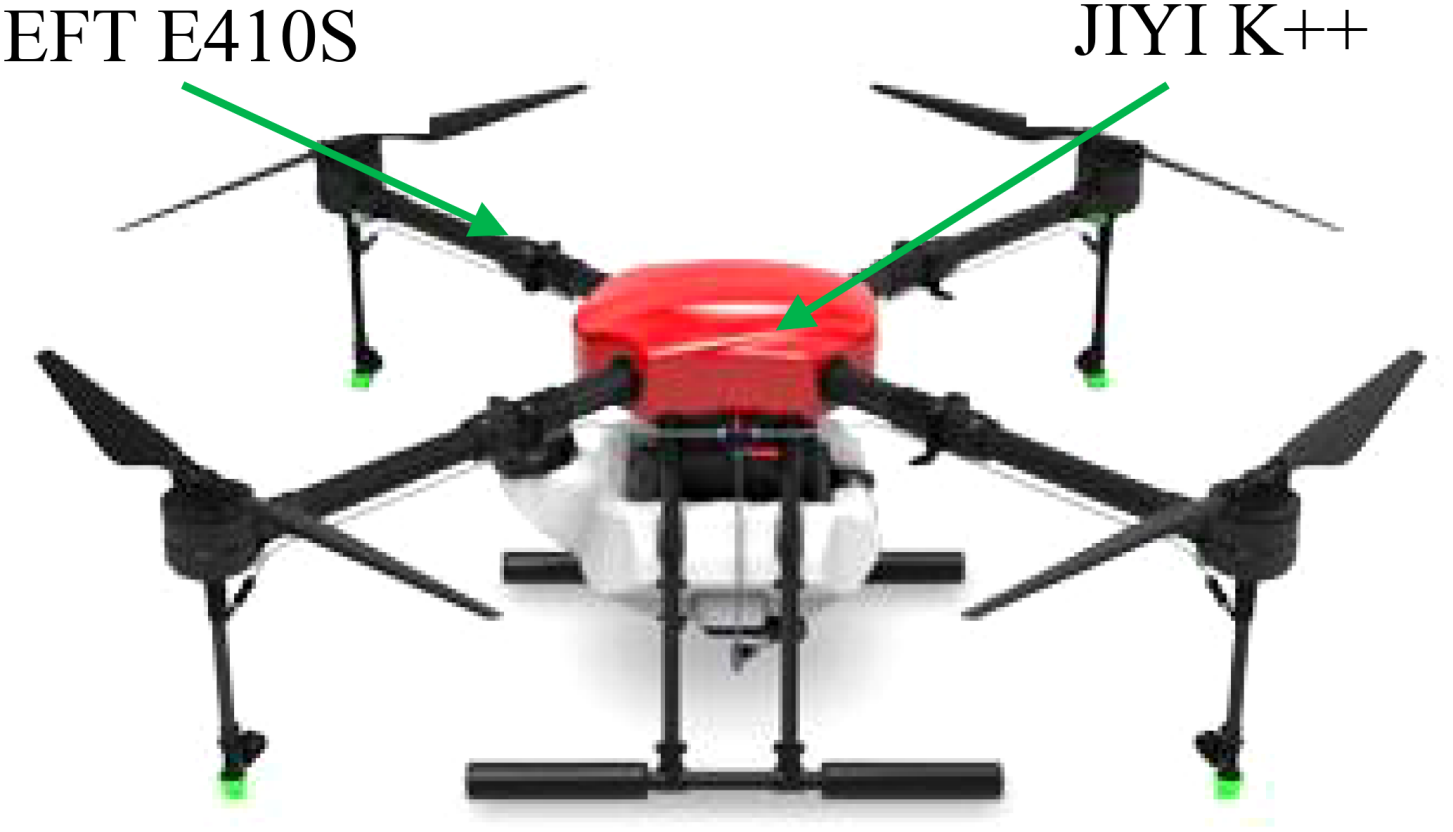
The structure diagram of the trichogramma balls UAV delivery system.

The trichogramma balls delivery system is built using a quad-rotor unmanned plant protection aircraft (EFT E410S) and a flight control system (JIYI K++). The structure is shown in **Figure 2**. The drone has a maximum operating height of 50m, a maximum speed of 10m/s, and a maximum takeoff weight of 25kg. The main frame is made of carbon fibre material. The folding arms are symmetrically mounted on the two diagonal lines of the frame using injection molding technology. A motor is installed at the end of the arm(Zhan et al., 2021; Ji et al., 2022).

**Figure 3:**
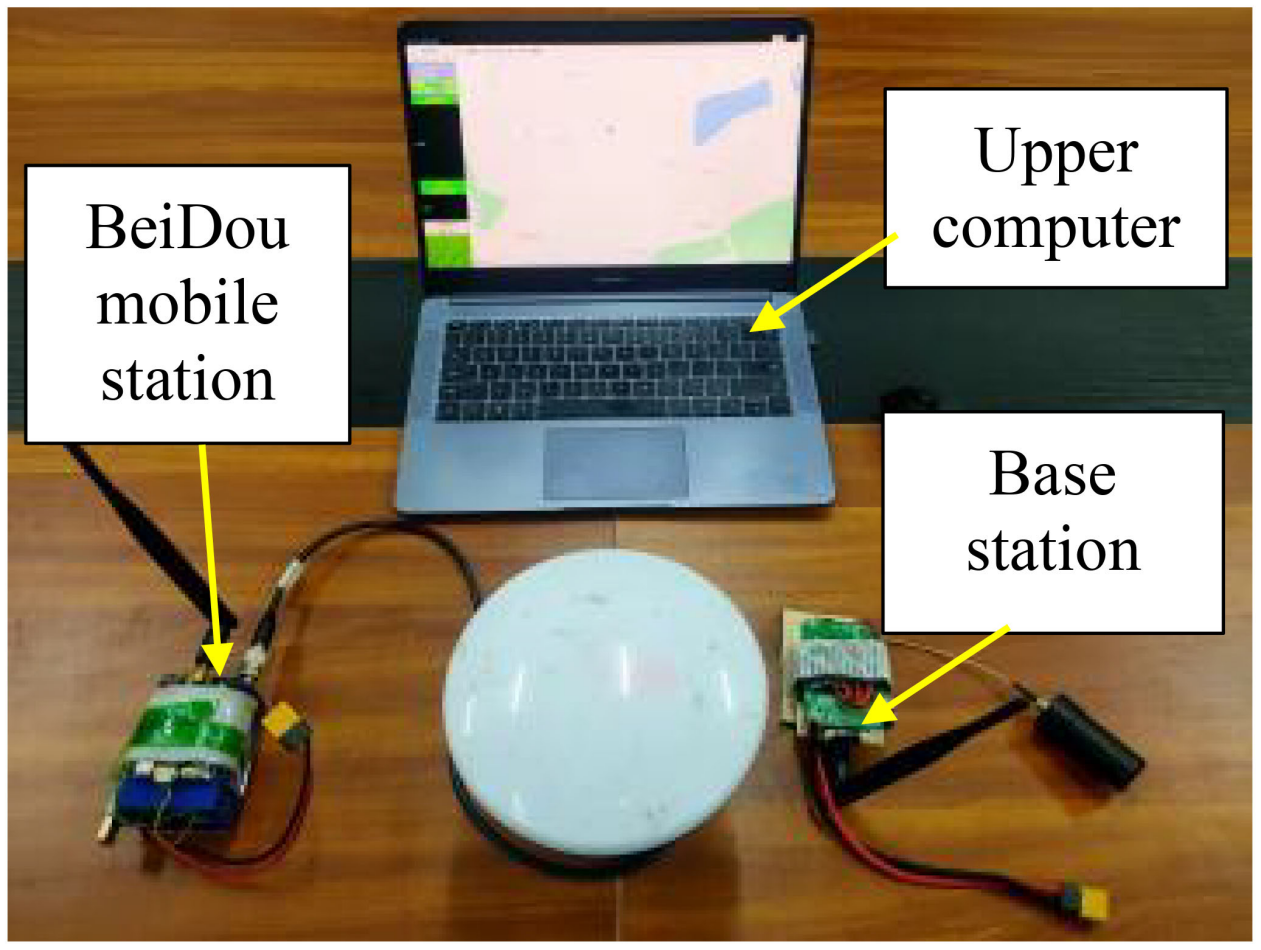
Ground control system.

The ground control system includes BeiDou mobile station, base station, and upper computer to obtain real-time information on flight altitude, speed and trajectory parameters of the drone during operation, as well as the actual landing point location information of the trichogramma balls. The structural diagram is shown in **Figure 3**. The core module of the system is the BeiDou board card (ComNav K705), with detailed technical parameters shown in **Table 1**. A data transmission module (Microhard P900) is used as the Beidou data wireless transmission device.

**Table 1:** Technical Parameters of K705 Board.

Technical parameter	Parameter value
Single point positioning accuracy	H<1.5 m, V<3 m (1δ, PDOP<4)
Static differential accuracy	H: ±(2.5 + 1×10^-6^×D) mm V: ±(5.0 + 1×10^-6^×D) mm
RTK accuracy	H: ±(8.0 + 1×10^-6^×D) mm V: ±(15 + 1×10^-6^×D) mm
Timing accuracy	20 *ns*

**Figure 4:**
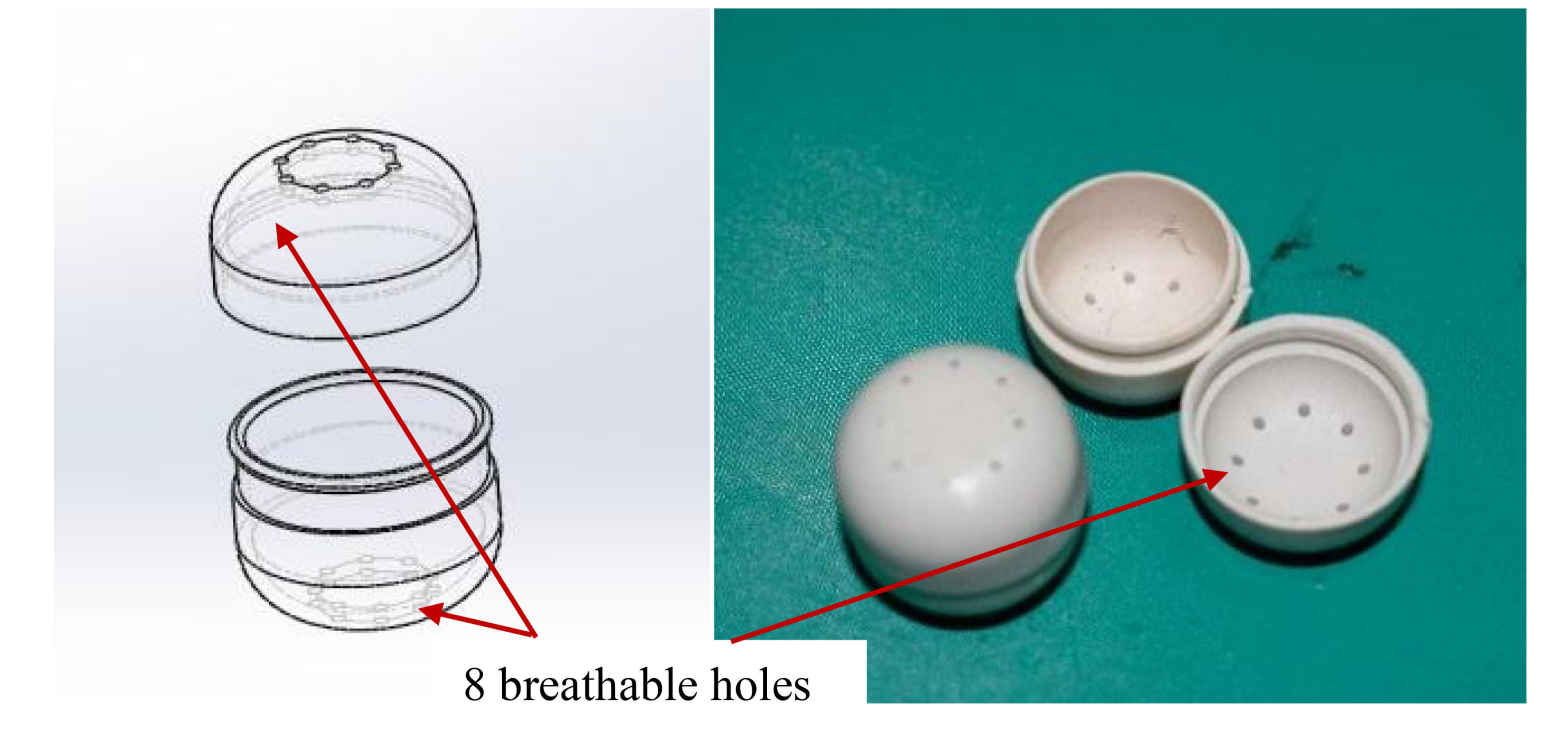
The structure of the trichogramma ball.

The trichogramma ball is made of degradable composite material using injection moulding technology. The trichogramma eggs are loaded inside the trichogramma ball, which can avoid the trichogramma from being exposed to the outside world before eclosion. The shape of the trichogramma ball is like a cylinder, with a diameter of 18 mm and a height of 18 mm, the edges of both ends are rounded with a radius of 5 mm, and the maximum diameter length is 20.5 mm, this structure can prevent the trichogramma ball from rolling too far after falling to the ground. There are eight breathable holes on both ends with a diameter of 0.5 mm, preventing natural enemies from swallowing insect eggs while ensuring a certain degree of breathability. The structure of the trichogramma ball is shown in **Figure 4**.

**Figure 5:**
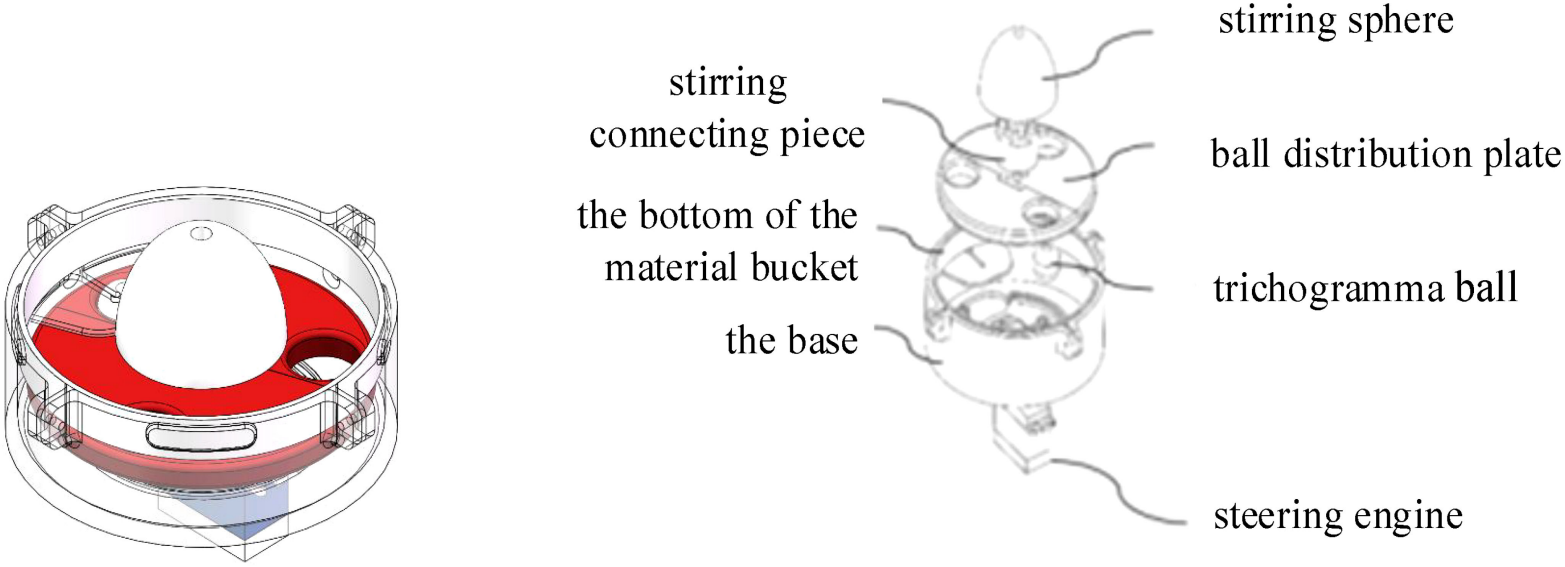
Delivery device structure.

The delivery device mainly includes a base, material bucket, balls distribution plate, stirring connecting piece, stirring sphere, and steering engine, as shown in **Figure 5**. The base matches the bottom of the material bucket, the four pits in the inner circle correspond to the protrusions around the bottom hole of the material bucket, two circular tracks are designed on the base, then the entering balls can slide along the tracks, ensuring that the balls can fall into the balls distribution plate at any posture. After the steering engine is installed on the base, the steering plate is sequentially connected with the balls distribution plate, the stirring connecting piece and the stirring sphere through bolts.

The circuit part of the control system of the trichogramma balls delivery device is composed of three parts: the power module (LM2596HVS step-down module), the control module (STM32F103C8T6), and the output module (DS3230 360° metal digital rudder). These components are directly connected to the UAV flight control system to realize the matching of the UAV flight speed and the trichogramma balls delivery speed. That is, the distance between trichogramma balls released at any flight speed is always a fixed value, so as to avoid the problem of uneven delivery of the balls in the field and achieve the linkage effect. The steering engine rotates when the PWM signal is input, so the speed of the steering engine is tested under different PWM input signals, and the functional relationship between the PWM signal value *x*_1_(*μs*) (input to the steering engine and the steering engine speed *n*(r/min) obtained by fitting is shown in Equation 1, the determination coefficient *R* ^2 ^= 0.9968.


(1) $$n=\frac{x_{1}-1495.5}{22.802}$$

The steering engine rotates 1/3 of a turn to drop a trichogramma ball and the UAV is required to put the trichogramma balls at equal intervals *l*(m), the UAV flight speed *v*(m/s) and the steering engine speed *n*(r/min) meet the Equation 2:


(2) $$n=\frac{1}{3}\frac{60v}{l}=\frac{20v}{l}$$

Therefore, the relationship between the PWM signal value *x*_1_(*μs*) input to the steering engine and the UAV flight speed *v*(m/s) can be expressed as follows:


(3) $$x_{1}=456.04\frac{v}{l}+1495.5$$

Through flight test, the relationship between PWM value *x*_2_(*μs*) of flight controller output and UAV flight speed *v*(m/s) can be obtained as follows:


(4) $$x_{2}=83.333v+1000$$

Then the PWM value *x*_2_(*μs*) of flight controller output and the PWM signal value *x*_1_(*μs*) input to the steering engine should meet the following relationship:


(5) $$x_{1}=\left(x_{2}-1000\right)\frac{5.473}{l}+1495.5$$

### 2.2 Performance test of the trichogramma balls delivery system

In the actual field operations of UAVs, the mechanical structure stability and control system accuracy of the trichogramma balls delivery system will directly affect the quality of field operations of the system. Therefore, experiments were conducted on system stability and release accuracy.

The test site was located at the Qilin District Sports Ground of South China Agricultural University (23.15°N 113.35°E). The test was conducted under the conditions of sunny weather, no wind, and 28°C temperature. Each time, 200 balls were placed in the material bucket, and five steering speeds of 6 m/s, 9 m/s, 12 m/s, 15 m/s and 18 m/s are set up through the delivery device control system. Three repeated releasing experiments are conducted respectively, and the number of balls released and the number of blocked and missed balls within two minutes were recorded, as shown in **Table 2**.

**Table 2:** Number of released balls.

Steering engine speed (r/min)	Number of balls released within 2 minutes				Total number of balls released	Total number of blocked and missed balls
	Group 1	Group 2	Group 3	precision (%)		
6	36	35	36	99.07%	200	0
9	53	54	54	99.38%	200	0
12	74	74	73	97.69%	200	0
15	91	92	92	98.15%	200	0
18	108	109	108	99.69%	200	0

As can be seen from **Table 2**, when the steering speed is 6 r/min, 9 r/min, 12 r/min, 15 r/min and 18 r/min, the device’s accuracy rate for releasing within 2 minutes reaches over 97.69%. Moreover, under these five speeds, all loaded balls are released without any issues of stuck balls or missed releases, indicating that the mechanical structure of this device is stable and reliable in releasing balls.

The trials conducted for the accuracy of system deployment in flight linkage mode includes two flight trajectories, each 80m long and 10m apart, with the UAV flight altitude set at 5m, UAV speeds are set to 3 m/s, 5 m/s, 7 m/s, and 9 m/s (Zhan et al., 2021). After each trial was completed, a tape measure was used to manually measure the distance between two adjacent balls on the same flight route (excluding acceleration and deceleration intervals). The actual landing site spacing data of the trichogramma balls under the four UAV flight speeds were recorded, and A One-way ANOVA (*α* = 0.05) was conducted using IBM SPSS (26.0) software. This study explored the impact of changes in UAV flight speed on the distance between the actual deployments of trichogramma balls, obtaining data in **Table 3** (Ding et al., 2021; Fang et al., 2022; Wang et al., 2022).

**Table 3:** Experimental and analytical data on the spacing of trichogramma balls placement.

Flight speed (m/s)	3		5		7		9	
	Route 1	Route 2	Route 1	Route 2	Route 1	Route 2	Route 1	Route 2
Spacing 1(m)	10.0	9.7	8.0	11.0	9.4	9.3	9.1	9.9
Spacing 2(m)	10.5	10.0	12.5	11.4	9.3	10.0	10.6	11.2
Spacing 3(m)	10.0	9.4	10.1	10.3	11.6	10.5	9.9	9.9
Spacing 4(m)	9.8	10.2	10.6	10.4	9.3	11.1	11.8	10.9
Spacing 5(m)	11.3	9.6	11.0	10.7	11.4	11.0	10.8	10.1
Spacing 6(m)	9.9	10.7	9.9	8.4	10.2	11.0	9.6	9.7
Average spacing(m)	10.3	10.0	10.4	10.4	10.2	10.5	10.3	10.3
Sum of squares	0.543							
Mean square	0.181							
F	0.231							
Sig.	0.874							

As can be seen from **Table 3**, when the test level is α = 0.05, the F test falls in the acceptance range, sig. = 0.874 > 0.05, which indicates that the change in UAV flight speed do not have a significant impact on the actual landing site spacing between adjacent balls. Moreover, as shown in **Table 3**, the average spacing between adjacent balls is between 10.0 m and 10.5 m at the above four flight speeds. This proves that UAV’s flight control can accurately coordinate with the delivery device to drop balls at the set spacing, and the release process is accurate and reliable.

### 2.3 Field operational quality evaluation test

**Figure 6:**
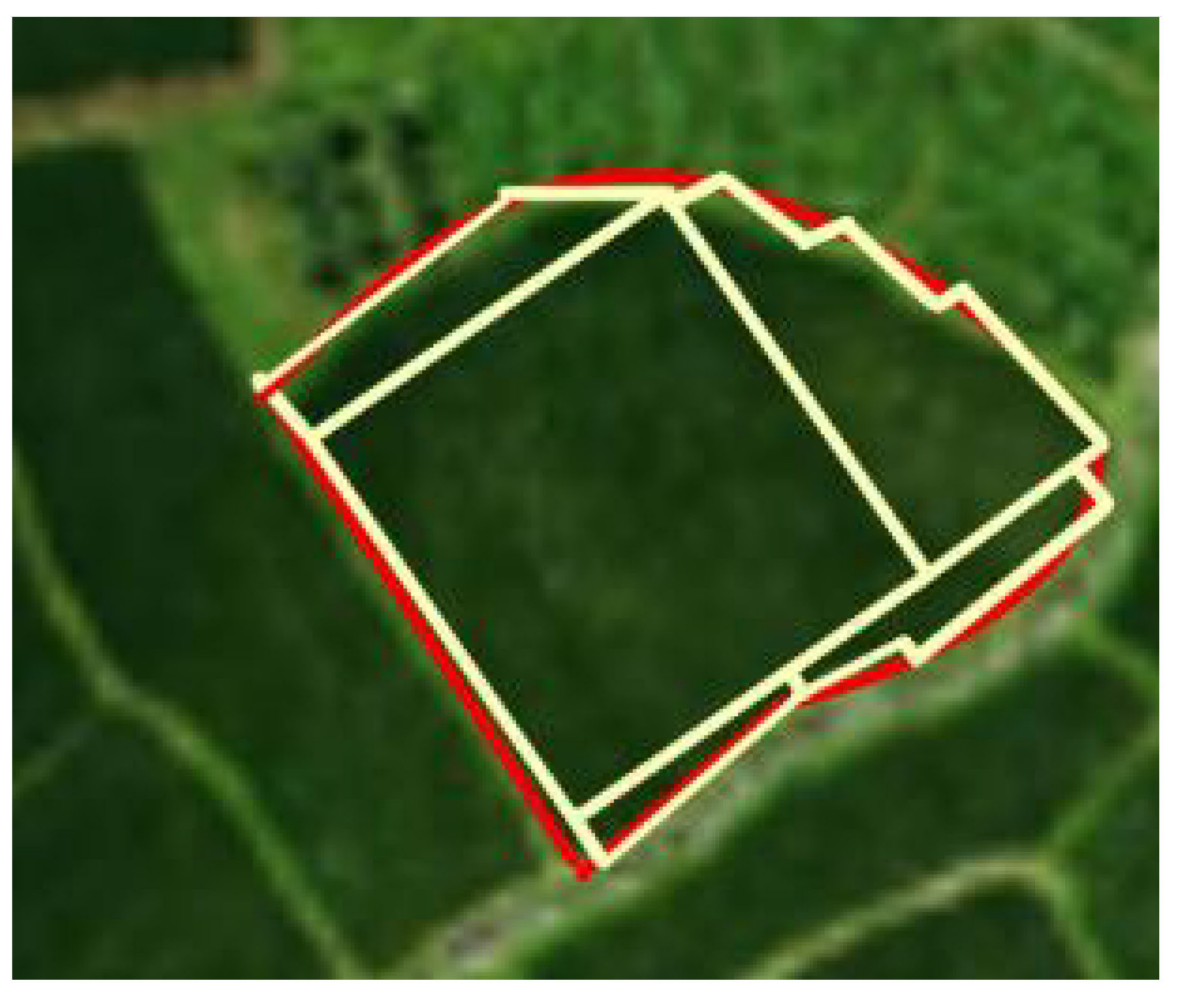
Natural field boundary diagram.

The natural crop fields in our country often present complex boundaries, as shown in **Figure 6**. If the red lines are used to indicate the shape of the complex boundary of the field in the figure, and the yellow lines are used to indicate the basic polygons in the field, it can be seen that except for the largest rectangular field in the center of the field, any irregular field at the boundary position can be deconstructed from rectangular, trapezoidal, and stepped fields. The operational quality at the boundary position is an important factor affecting the overall operational quality of the entire field.

**Figure 7:**
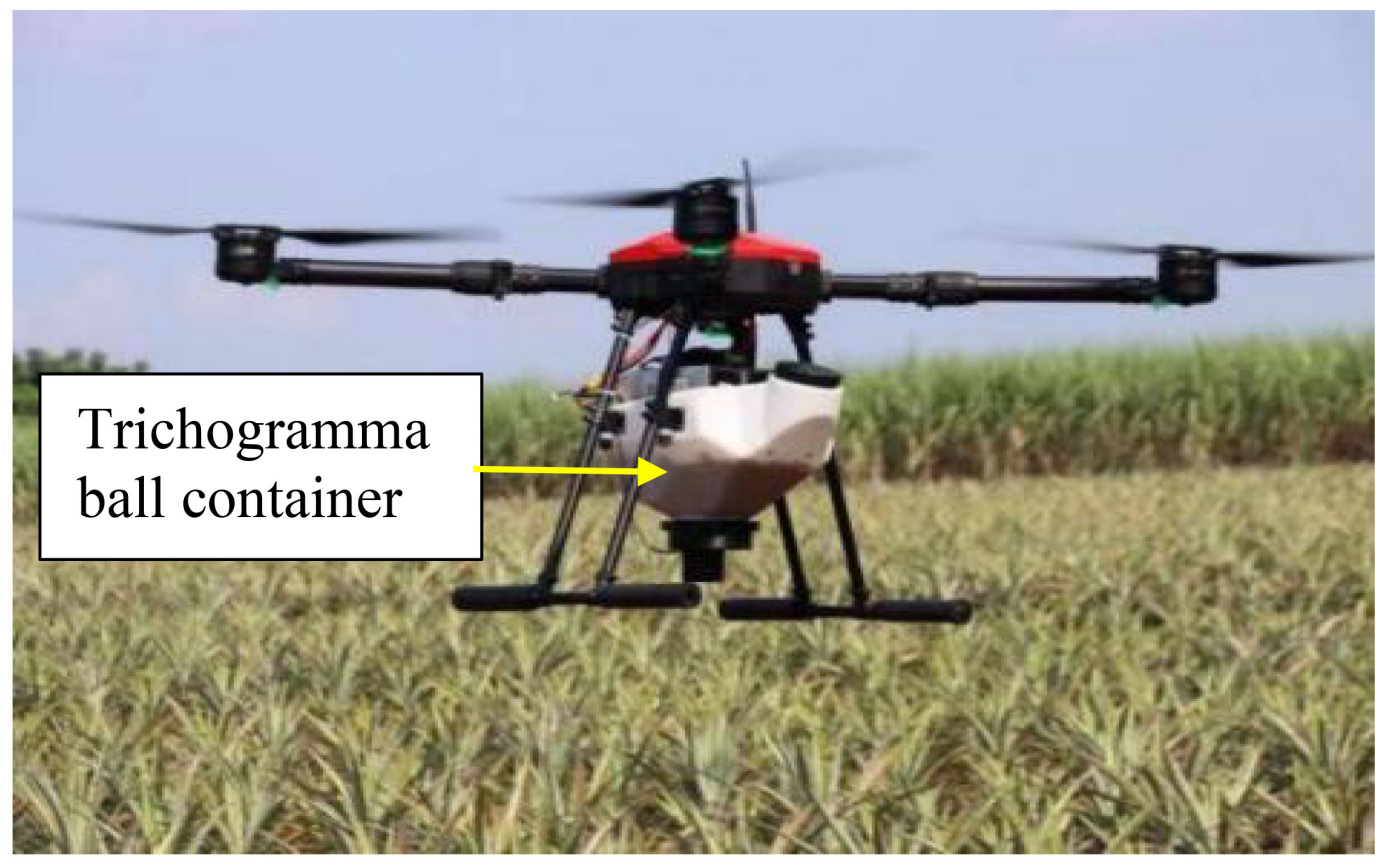
Field test implementation of the system.

**Figure 8:**
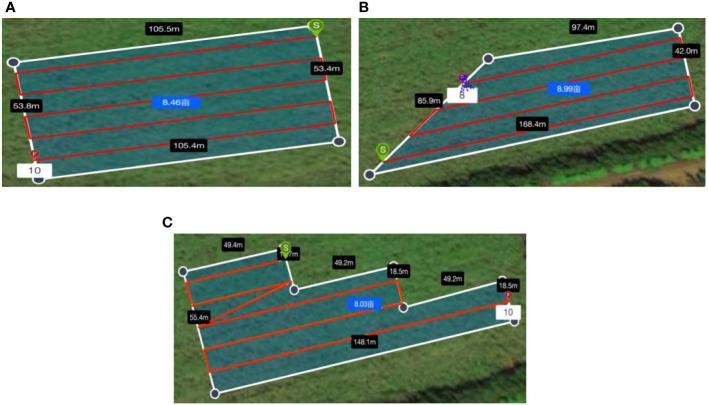
Schematic diagram of field plots in field experiments.

To explore the influence of rectangular, trapezoidal and stepped boundary fields on the overall field operational quality, a field operation test of the trichogramma balls delivery system was carried out, as shown in **Figure 7**. The test site was located in a pineapple field at Guangken Agricultural Machinery in Zhanjiang, Guangdong (21.3°N 110.3°E). The rectangular, trapezoidal, and stepped fields with an area of approximately 8 acres were selected. The actual field area and boundary size of the three test plots were measured and marked on **Figure 8**, where the white line frame represented the boundary of the operational area, the red line represented the flight route of the UAV, the green circle marked with *S* represented the starting point of the UAV for releasing the balls, and the red circle marked with *F* represented the ending point of the UAV (Cao et al., 2020).

**Figure 9:**
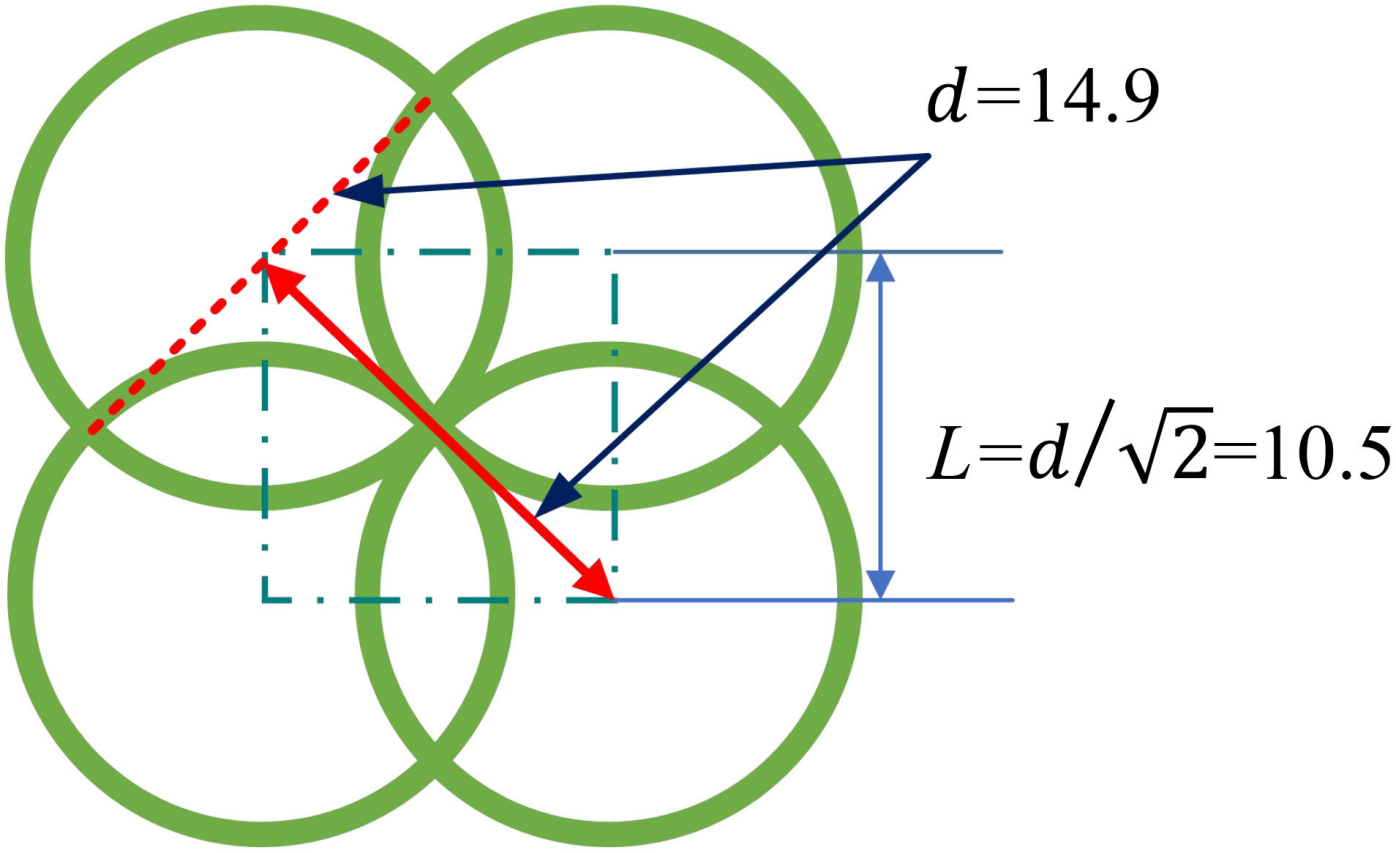
Trichogramma balls effect coverage area.

**Figure 10:**
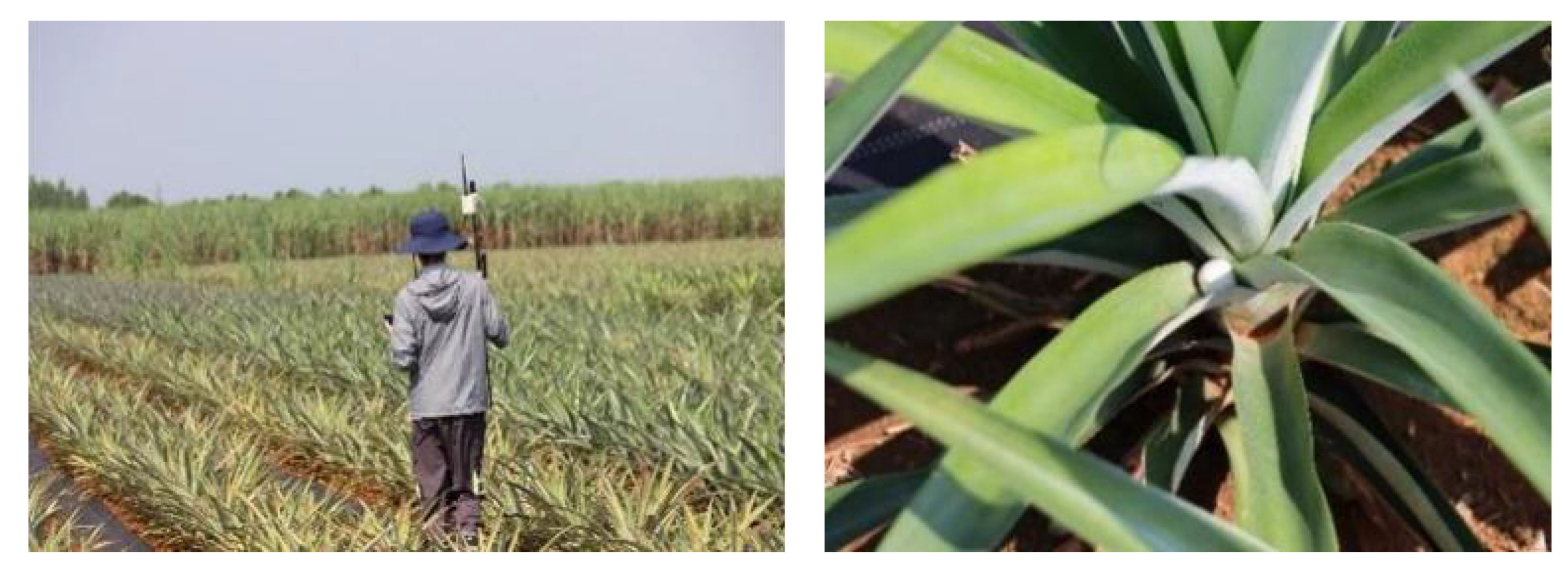
Manual sampling of trichogramma ball drop points data.

A trichogramma ball stores approximately 2000 trichogramma eggs, and the range of protection after the hatching of the contained trichogramma eggs is a circle with a diameter of 14.9 m centred on the trichogramma ball, as shown in the green circle in **Figure 9**. To achieve full coverage of the UAV operational area, the spacing *L* of the released balls should be less than or equal to 10.5 m , $\left(\text{d}/\sqrt{2}\right)$ as shown in **Figure 9**, so the flight path spacing is set to 10.5 m. The flight speed is set to 7 m/s. After the test flight is completed, the mobile station is removed from the UAV and installed on a handheld fixture to make a ball landing point data sampler. The locations of the dropped balls were sampled one by one in the pineapple field, as shown in **Figure 10**.

### 2.4 Field operational quality evaluation method

**Figure 11:**
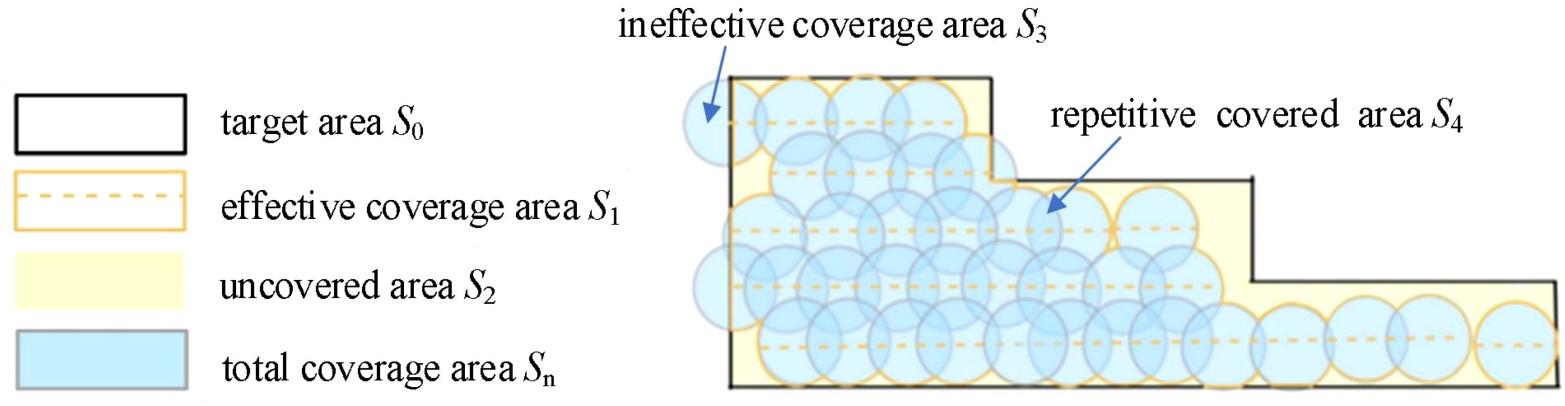
Schematic diagram of area indicators.

In order to evaluate the actual field operational quality of the trichogramma balls UAV delivery system in linkage mode, an actual landing map was drawn based on the actual operational route and the landing point position, that is, the actual operational status was drawn in Auto CAD (2022). The target area *S*_0_, effective coverage area *S*_1_, uncovered area *S*_2_, ineffective coverage area *S*_3_, repetitive covered area *S*_4_, and total coverage area *S_n_* are defined as shown in **Figure 11**, and the operational performance indicators, effective coverage rate *η*_1_, uncovered rate *η*_2_, ineffective coverage rate η_3_, utilization rate of balls *η*_4_, and repetition rate of balls *η*_5_, are also defined as follows.

The effective coverage rate *η*_1_ meets Equation 6:


(6) $$\eta_{1}=S_{1}/S_{0}$$

The uncover rate *η*_2_ meets Equation 7:


(7) $$\eta_{2}=S_{2}/S_{0}$$

The ineffective coverage rate *η*_3_ meets Equation 8:


(8) $$\eta_{3}=S_{3}/S_{0}$$

The utilization rate of balls *η*_4_ meets Equation 9:


(9) $$\eta_{4}=S_{1}/(S_{0}+S_{3})$$

The repetition rate of balls *η*_5_ meets Equation 10:


(10) $$\eta_{5}=S_{4}/S_{n}$$

The system operational indicators *η*_1_,*η*_2_,*η*_3_,*η*_4_,*η*_5_, all affect the overall operational quality of the system, but the influence degrees of each are different, so the influence of each indicator needs to be reflected by weights. The entropy weight method is an objective weighting method for determining the weights of evaluation indicators. This method is based on the current sample data to obtain the weight of the indicators by statistics, avoiding deviations caused by human factors. The process of evaluating the overall operational quality using the entropy weight method is as follows (Qin et al., 2023):

1) Construct an evaluation indicator matrix $X={\left(x_{ij}\right)}_{mn},i=1,2,\cdots ,m;j=1,2,\cdots ,n$ . where *x_ij_* represents the value of *j*-th indicator of the *i*-th sample. *m* represents the number of field types, and *n* represents the number of indicators.

2) Standardize the indicators. If the indicator has a positive effect (the larger, the better), the standardization method is as follows.


(11) $$x_{ij}^{'}=\frac{x_{ij}-minx_{ij}}{maxx_{ij}-minx_{ij}},i=1,2,\cdots ,m;j=1,2,\cdots ,n$$

If the indicator has a negative effect (the smaller, the better), the standardization method is as follows.


(12) $$x_{ij}^{'}=\frac{maxx_{ij}-x_{ij}}{maxx_{ij}-minx_{ij}},i=1,2,\cdots ,m;j=1,2,\cdots ,n$$

Where $x_{ij}^{'}$ is the standardized indicator value and represents the *j*-th standard indicator value of the *i*-th sample.

3) Use the translation method to handle special situations that may occur after standardization.


(13) $$x_{ij}^{''}=H+x_{ij}^{'},i=1,2,\cdots ,m;j=1,2,\cdots ,n\;H=0.01$$

4) Calculate the proportion matrix *P* = (*p_ij_*)*_mn_*, where *p_ij_* represents the proportion of the *j*-th indicator value of the *i*-th sample, and take it as the probability when calculating the information entropy.


(14) $$p_{ij}=\frac{x_{ij}^{''}}{\sum_{i=1}^{m}x_{ij}^{''}},i=1,2,\cdots ,m;j=1,2,\cdots ,n$$

5) Calculate the information entropy matrix of the evaluation indicators *E* = (*e_j_*). *e_j_* represents the information entropy of the *j*-th indicator.


(15) $$e_{j}=\frac{1}{\text{ln}m}\sum_{i=1}^{m}p_{ij}I_{ij},j=1,2,\cdots ,n$$

Where $I_{ij}=-lnp_{ij}$ represents the information of the *j*-th indicator value of the *i*-th sample. The larger *p_ij_* is, the smaller the uncertainty is, and the less information it contains. The maximum value of $\sum_{i=1}^{m}p_{ij}I_{ij}$ is *ln*m, so dividing $\sum_{i=1}^{m}p_{ij}I_{ij}$ by a constant *ln*m to make *e_j_* falls between [0,1].

6) Calculate the evaluation matrix *W* = (*w_j_*). *w_j_* represents the weight of the *j*-th indicator.


(16) $$w_{j}=\frac{g_{j}}{\sum_{j=1}^{n}g_{j}},j=1,2,\cdots ,n$$

Where $g_{j}=1-e_{j}$ represents the information utility of the *j*-th indicator value. The larger the information entropy of the indicator, the less information it carries, and the smaller the information utility.

7) According to the objective weights obtained using the entropy weight method, the comprehensive score is calculated using the linear weighting method for overall operational quality evaluation.


(17) $$F_{i}=\sum_{j=1}^{n}w_{j}x_{ij}^{'},i=1,2,\cdots ,m;j=1,2,\cdots ,n$$

## 3 Results and discussion

### 3.1 Ideal and actual delivery operational effects

**Figure 12:**
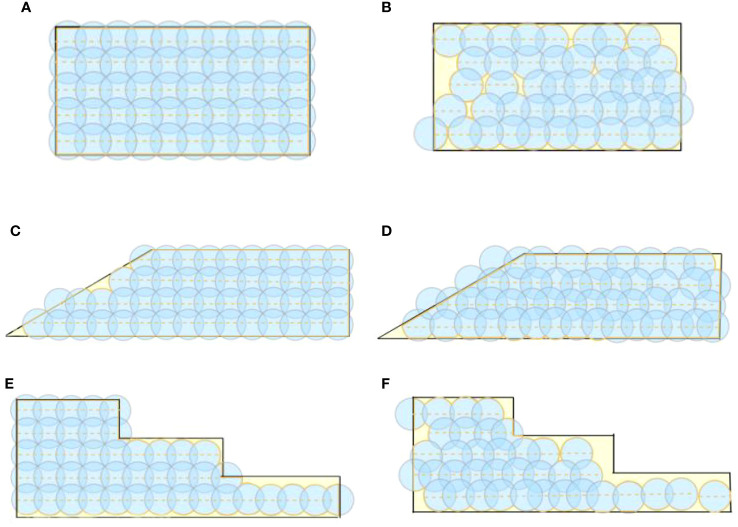
Schematic diagram of the trichogramma balls coverage range: **(A)** Ideal coverage diagram of rectangular fields, **(B)** Actual coverage diagram of rectangular fields, **(C)** Ideal coverage diagram of trapezoidal fields, **(D)** Actual coverage diagram of trapezoidal fields, **(E)** Ideal coverage diagram of stepped fields, **(F)** Actual coverage diagram of stepped fields.

In the ideal operational state, without considering factors such as flight route deviation, the ideal landing point diagram of the trichogramma ball is obtained. Using the center of the trichogramma ball’s ideal landing point as the center of a circle *d* = 14.9 m, a schematic diagram of the coverage of the balls in three types of fields is drawn as shown in **Figures 12A, C, E**. The data is analyzed to obtain the ideal operational data in **Table 4**.

**Table 4:** Operational data of the trichogramma balls delivery system.

	Ideal Operation			Actual Operation		
	rectangle	trapezoidal	stepped	rectangle	trapezoidal	stepped
Number of pitches	50	50	44	45	50	37
*S_n_* (m²)	6030.16	6156.98	5522.14	5451.53	6209.67	4793.63
*S*_0_ (m²)	5565.00	5565.00	5565.00	5565.00	5565.00	5565.00
*S*_1_ (m²)	5564.11	5495.21	5135.73	5192.61	5419.44	4488.89
*S*_2_ (m²)	0	64.14	438.53	372.39	145.56	1076.11
*S*_3_ (m²)	465.16	656.10	251.84	258.92	776.13	303.20
*S*_4_ (m²)	2694.95	2568.13	2155.96	2401.07	2515.44	1662.95
*η*_1_ (%)	99.98	98.74	92.28	93.31	97.38	80.66
*η*_2_ (%)	0	1.15	7.88	6.69	2.61	19.33
*η*_3_ (%)	8.36	11.79	4.52	4.65	13.94	5.45
*η*_4_ (%)	92.27	88.33	88.29	89.16	85.46	76.49
*η*_5_ (%)	44.69	41.71	39.04	44.04	40.51	34.69
Total pitching time (*s*)				125.00	125.80	126.20
Area delivered per unit time (m²/min)				3766.39	4158.27	3067.36

**Figure 13:**
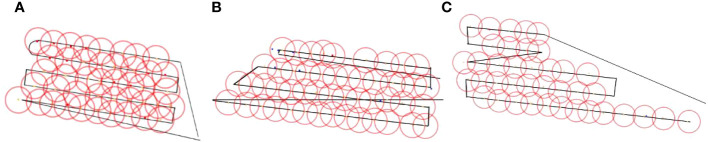
Schematic diagram of the actual coverage range: **(A)** Rectangular field, **(B)** Trapezoidal field, **(C)** Stepped field.

In the actual experimental field, the lush foliage of pineapple branches and the small size of the trichogramma ball make it very easy for falling balls to go unnoticed, resulting in a lack of actual landing points or redundant sampling due to previous missed detection. To avoid these situations affecting the experimental data, an average spacing is used as a reference, for obvious redundant landing points along the flight path, ideal ball landing points are used as reference points for deletion. for obvious missing points, including obvious missing points at the beginning or end of the route, the data is filled at an average spacing from the nearby reference point(the nearby actual landing point). Then With the landing point as the center of the circle *d* = 14.9m, a schematic diagram of the effective coverage area is drawn, as shown in **Figure 13**, which is simplified to **Figures 12B, D, F**. The actual operational parameters and performance indicators are also shown in **Table 4**. In terms of the uniqueness of the test results, as can be seen from **Figures 12A, C, E**, to ensure full coverage of the target area in rectangular, trapezoidal, and stepped fields, the flight path and release points are unique. Therefore, in the operation, the starting point of the UAV to begin to drop the trichogramma balls needs to be marked in advance in the flight control system.

From **Table 4**, it can be seen that in actual delivery operations, only the number of balls dropped in trapezoidal fields (50) reaches the ideal number of delivery operations (50), while rectangular (45) and stepped (37) fields do not achieve the same ideal number of delivery operations (50, 44), which will have an impact on the operational effect. In actual operations, the coverage rate of balls dropped can reach 97%, and the delivery area per unit time can reach 4158 m²/min.

**Figure 14:**
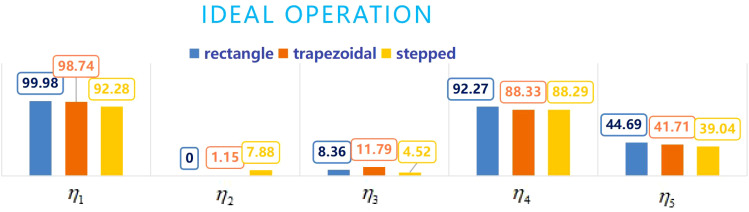
Comparison chart of five indicators for three fields under ideal operating conditions.

**Figure 15:**
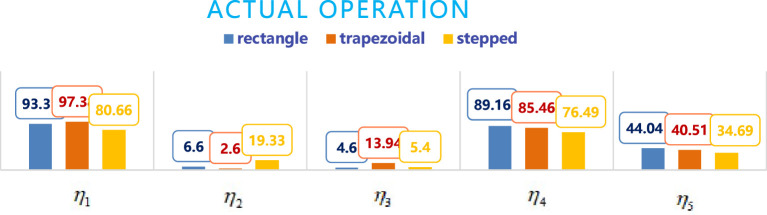
Comparison chart of five indicators for three fields under actual operating conditions.

From the data in **Table 4**, the comparison chart of the five indicators obtained from three different boundary fields under ideal and actual operating conditions is shown in **Figures 14** and **15**, respectively. From **Figures 14** and **15**, it can be seen that in terms of the effective coverage rate *η*_1_, in ideal situations, rectangle *η*_1_ > trapezoidal *η*_1_ > stepped *η*_1_; in actual situations, trapezoidal *η*_1_ > rectangle *η*_1_ > stepped *η*_1_, stepped *η*_1_ is always the smallest. The main reason is that the height of the ladder at the step is not up to the height standard of two trichogramma balls, when planning the path, it can only be covered by one trichogramma balls delivery route, and the number of pitches is minimum, which leads to the increase of the uncovered area of the operation and the decrease of the effective coverage rate *η*_1_. For rectangular and trapezoidal fields, under ideal operational conditions, all four sides of the rectangular field are regular right-angled sides (90°), the positioning of the pitch points is planned so that the trichogramma balls can cover all routes and also cover all target area. For the trapezoidal field, at its hypotenuse, although the planned pitch point can cover all routes, it does not consider the difficult-to-cover gaps between upper and lower flight routes caused by the oblique edges, therefore, in the ideal situations, rectangle *η*_1_ > trapezoidal *η*_1_. In actual situations, trapezoidal *η*_1_ > rectangle *η*_1_, and compared to the ideal situations, the overall *η*_1_ declined. The main reason for this is that during actual operations, deviations in drone flight paths and the landing positions of the trichogramma balls lead to a decline in the overall coverage effects. It is also found that during actual operations, the largest uncovered area is at the edges and corners of the field.

**Figure 16:**
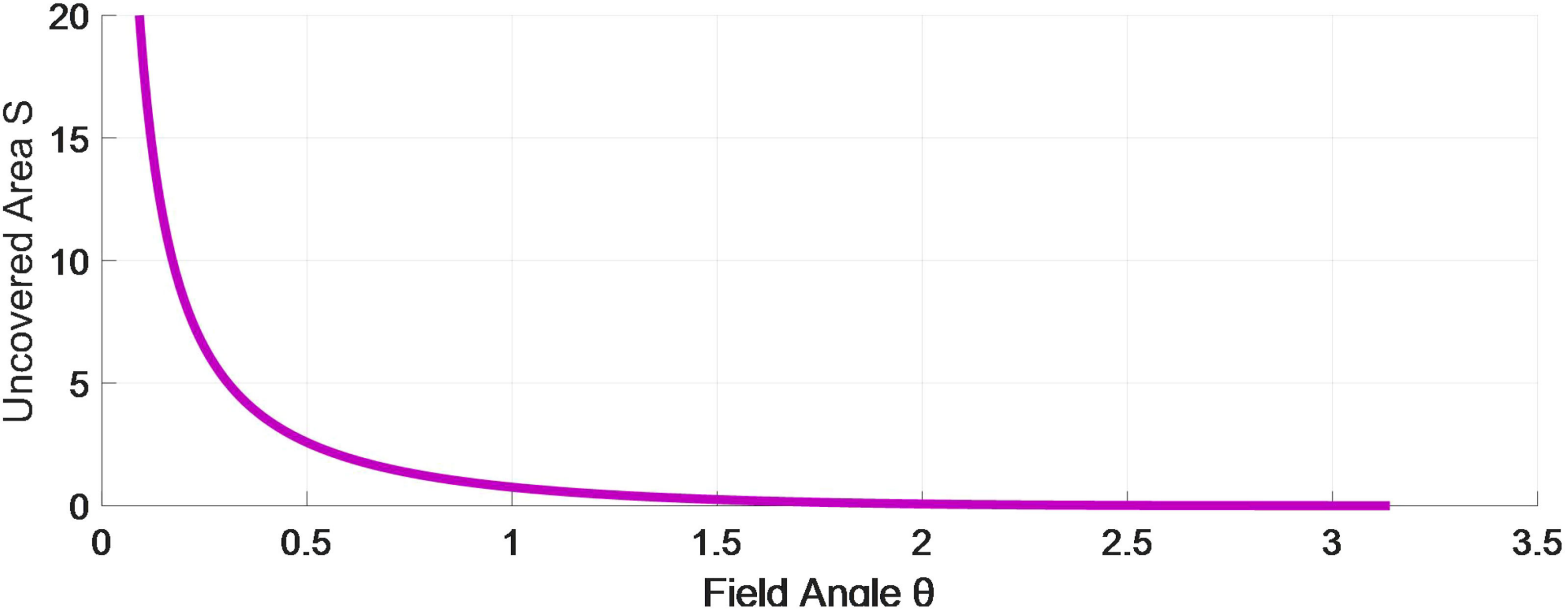
Relationship Curve between Field Angle *θ* and Uncovered Area *S*.

**Figure 17:**
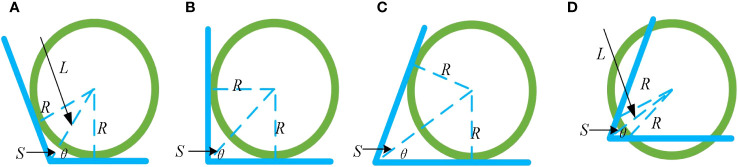
Diagram of the fit relationship between edges and circles: **(A–D)**.


Theoretically, the relationship between the field edge angle *θ* and the uncovered area *S* at the edge corners satisfies Equation 18, and its relationship curve is shown in **Figure 16** (when *R*=1). The schematic diagram is shown in **Figure 17**.

(18) $$S=R^{2}\left[tan\frac{\pi -\theta }{2}-\frac{\pi -\theta }{2}\right]$$

As shown in **Figure 16**, with the increase of *θ*, the uncovered area decreases, and the circular area covered by the ball increasingly matches the edge boundaries. Relatively speaking, when *θ* > 90° (**Figure 17A**), the circular area covered by the ball is more fit with the boundary. The larger *θ* is, the higher the fit and the smaller the uncovered area are. When *θ* = 90° (**Figure 17B**), the circular area covered by the ball is difficult to fit with the boundary, and the uncovered area is larger than that when *θ* > 90°. When *θ* < 90° (**Figure 17C**), the circular area covered by the ball is the most difficult to fit with the boundary, and the uncovered area is larger than that when *θ* = 90°. Since the extension of the circular area covered by the balls beyond the field boundary does not bring about any damage similar to that after pesticide spraying, it only leads to an increase in the ineffective coverage area of the trichogramma balls.

Therefore, considering that the distance between the balls release point and the vertex of the edge and corner should be equal to that between the balls release point and vertex of edge and corner in **Figure 17A**), both are *L* (**Figures 17A, D**), so as to reduce the large uncovered area caused in **Figure 17C**. Therefore, in actual operations, this system can easily achieve a higher effective coverage rate *η*_1_ in fields with *θ* > 90°, and the effective coverage rate *η*_1_ is the lowest in stepped fields due to they have more right-angle edges.

Due to the fact that the sum of effective coverage rate *η*_1_ and uncover rate *η*_2_ is 1, the changes of *η*_1_ and *η*_2_ in the three types of fields exhibit an inverse synchronization relationship. Therefore, when quantifying the effectiveness of the operation, one of the changes is selected as an evaluation indicator. In this operational quality evaluation system, the effective coverage rate *η*_1_ is chosen as the system operational effect evaluation indicator.

In terms of the invalid coverage rate *η*_3_, it is clear that the lower the *η*_3_, the better the operation. From **Figures 14** and **15**, it can be seen that ideally, the stepped *η*_3_< rectangle *η*_3_< trapezoidal *η*_3_. Actually, rectangle *η*_3_<stepped *η*_3_< trapezoidal *η*_3_,with the trapezoid always being the largest, This is mainly due to that in order to improve the effective coverage area, the trapezoidal field adds the dropping point of the balls at the hypotenuse, thereby increasing the ineffective coverage area outside the target area. In ideal situations, stepped *η*_3_ < rectangle *η*_3_, mainly because the stepped field’s step height is less than the height required to put two balls, therefore, only one row of delivery points is planned, so that the invalid coverage area S_3_ is reduced. In actual situations, the rectangle *η*_3_ < stepped *η*_3_, mainly due to the deviation of the route and the position of the landing point at the right angle turning point, and at the right angle corners, this deviation is more pronounced, such as the stepped shape has multiple right angle corners, resulting in a greater pitch deviation and resulting in a smaller effective coverage area and a larger ineffective coverage area. However, ineffective coverage for the balls delivery operation does not lead to pesticide drift into invalid coverage areas as when spraying pesticides, so the influence of *η*_3_ can be weakened or even not considered in the evaluation process. The effectiveness of ineffective coverage is indirectly reflected by the utilization rate *η*_4_, obviously, the higher the better. Under ideal and actual operating conditions, all are rectangular *η*_4_ > trapezoidal *η*_4_ > stepped *η*_4_, but in terms of the balls repetition rate *η*_5_, obviously the lower the better. Under ideal and actual operating conditions, all are stepped *η*_5_ < trapezoidal *η*_5_ < rectangular *η*_5_, so it is difficult to simply judge which type of field is the best from the trichogramma balls utilization effect.

### 3.2 Analysis of operational quality evaluation

To quantify the differences in operational quality among three shapes of fields in ideal and actual delivery operations, we set up a comprehensive evaluation system for operational quality, It quantitatively analyzes from two aspects: the effectiveness of field operation *U*_1_ and the utilization effect of trichogramma balls *U*_2_. The aforementioned evaluation indicators are divided into two levels. The effective coverage rate *η*_1_ reflects the system operational effect *U*_1_, while the balls utilization rate *η*_4_ and repetition rate *η*_5_ reflect the trichogramma balls utilization effect *U*_2_. The specific grading is shown in **Table 5**.

**Table 5:** Comprehensive evaluation indicators and weights of operational quality.

primary indicators	weight (W)		secondary indicators	weight (W)	
Field operational effect (*U*_1_)	ideal	0.4092	*η*_1_	ideal	0.4092
				actual	0.5008
	actual	0.5008	*η*_2_	ideal	0
				actual	0
Trichogramma balls utilization effect (*U*_2_)	ideal	0.5908	*η*_3_	ideal	0
				actual	0
			*η*_4_	ideal	0.6966
	actual	0.4992		actual	0.4512
			*η*_5_	ideal	0.3034
				actual	0.5488

**Figure 18:**
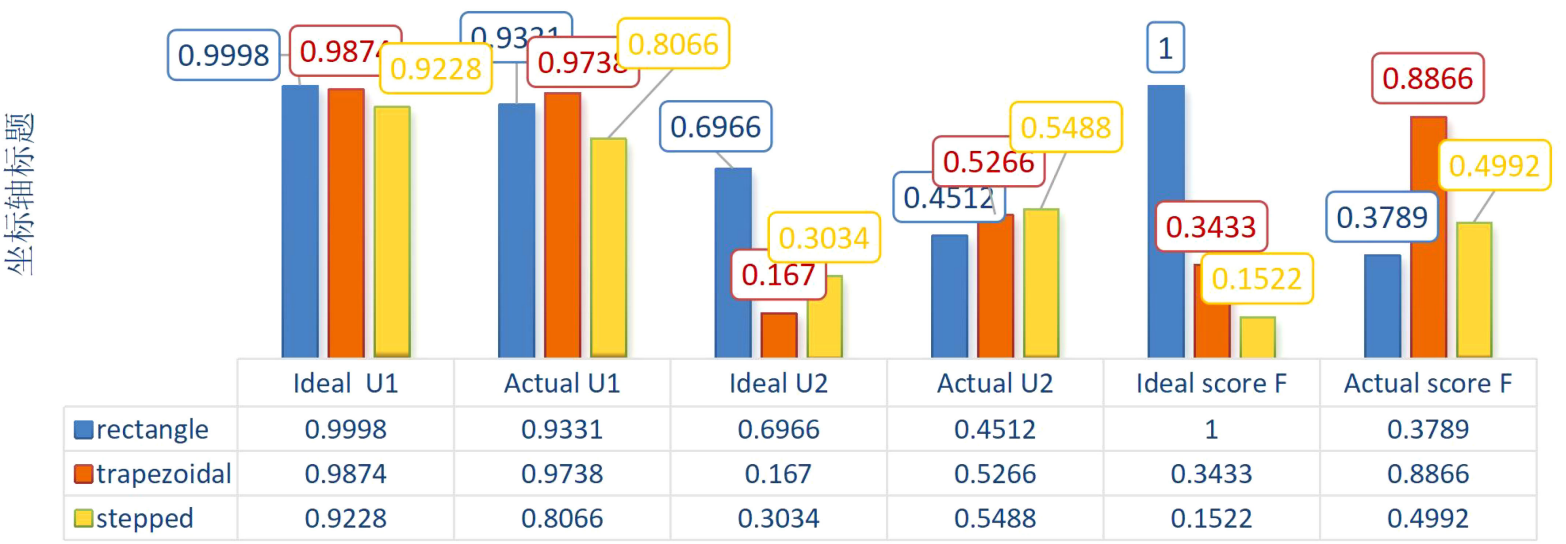
Comprehensive operational quality evaluation data of the three types of boundary fields.


Firstly, the entropy weight method is used to determine the secondary indicator weights of *η*_4_ and *η*_5_ to evaluate the utilization effect of trichogramma balls. The trichogramma balls utilization rate *η*_4_ is a positive effect indicator, the larger the indicator, the better the utilization effect of balls, which can be directly standardized using Equation 8. The repetition rate of the balls *η*_5_ is a negative effect indicator, the larger the indicator, the worse the utilization effect of balls, which should be converted into a positive effect indicator using Equation 9 to obtain the standardized matrix *X*_1_. Then, after translation processing using Equation 10, the proportion matrix *P*_1_ of *η*_4_, *η*_5_ is calculated according to Equation 11 for evaluating different fields, and the information entropy matrix *E*_1_ of *η*_4_, *η*_5_ is calculated according to Equation 12, the weight matrix *W*_1_ of *η*_4_, *η*_5_ is calculated according to Equation 13. The linear weighting method of Equation 14 is used to calculate the data of trichogramma balls utilization effect *U*_2_, and then the same method is used to determine the *X*_2_, *P*_2_, *E*_2_, and *W*_2_ of the two primary indicators *U*_1_, *U*_2_,The specific data obtained under ideal and actual conditions are as follows, and the indicator weights are shown in **Table 5**. The comprehensive field operational scores of three types of field blocks under ideal and actual conditions are obtained by using the linear weighting method with weights and standardized sample values, and the data are shown in **Figure 18**.

$$\begin{array}{ccc} X_{1ideal}=\left[\begin{array}{cc} 1.0000 & 0.0000\\ 0.0101 & 0.5274\\ 0.0000 & 1.0000 \end{array}\right] & P_{1ideal}=\left[\begin{array}{cc} 0.9881 & 0.0007\\ 0.0109 & 0.3453\\ 0.0010 & 0.6541 \end{array}\right] & E_{1ideal}=\left[\begin{array}{cc} 0.0618 & 0.5913 \end{array}\right] \end{array}$$

$$\begin{array}{ccc} X_{2ideal}=\left[\begin{array}{cc} 1.0000 & 1.0000\\ 0.8390 & 0.0000\\ 0.0000 & 0.2576 \end{array}\right] & P_{2ideal}=\left[\begin{array}{cc} 0.5434 & 0.7941\\ 0.4560 & 0.0007\\ 0.0005 & 0.2051 \end{array}\right] & E_{2ideal}=\left[\begin{array}{cc} 0.6313 & 0.4676 \end{array}\right] \end{array}$$

$$\begin{array}{ccc} X_{1actual}=\left[\begin{array}{cc} 1.0000 & 0.0000\\ 0.7080 & 0.3775\\ 0.0000 & 1.0000 \end{array}\right] & P_{1actual}=\left[\begin{array}{cc} 0.5850 & 0.0007\\ 0.4144 & 0.2741\\ 0.0006 & 0.7251 \end{array}\right] & E_{1actual}=\left[\begin{array}{cc} 0.6217 & 0.5399 \end{array}\right] \end{array}$$

$$\begin{array}{ccc} X_{2actual}=\left[\begin{array}{cc} 0.7566 & 0.0000\\ 1.0000 & 0.7228\\ 0.0000 & 1.0000 \end{array}\right] & P_{2actual}=\left[\begin{array}{cc} 0.4305 & 0.0006\\ 0.5689 & 0.4357\\ 0.0006 & 0.5637 \end{array}\right] & E_{2actual}=\left[\begin{array}{cc} 0.6262 & 0.6274 \end{array}\right] \end{array}$$

From **Figure 18**, it can be seen that from the perspective of system field comprehensive operational quality evaluation, under ideal operational conditions, the comprehensive operational quality score F of rectangular fields is the highest, reaching 1.000, while under actual operational conditions, the comprehensive operational quality score F of trapezoidal fields is the highest, at 0.8866. This indicates that in actual operations, if any complex boundary natural fields are decomposed into several rectangular, trapezoidal, or stepped small fields, the more trapezoidal small fields, the higher the comprehensive operational quality of this complex boundary field.

## 4 Conclusion

The initial use of chemical pesticides can effectively control pests, but long-term or excessive use of chemical pesticides can cause pollution of the atmosphere and soil, as well as harm to human health. Studies have shown that using natural enemies for biological pest control presents significant potential and can overcome the problems associated with chemical pesticide control. The introduction of trichogramma is currently a prevalent method of biological pest control. The deployment of trichogramma balls by UAVs can operate in various complex environments, offering advantages such as higher efficiency, faster speed, reduced costs, less waste, and fewer labor resources. However, current research on the trichogramma balls delivery system is still in its nascent stages. Especially when operating in fields with complex boundaries, the reliability and accuracy of this method need improvement, and the evaluation indicators for its effectiveness also need to be clarified. These issues are studied in this article. The experiment shows that the pitching accuracy of the trichogramma balls delivery system designed in this article exceeds 97.69%, and all loaded balls are released without any issues of stuck balls or missed releases, indicating that the system is accurate and reliable. The efficiency of the system can reach 4158 m^2^/min, and the coverage rate of trichogramma balls can exceed 97%.

In order to comprehensively evaluate the actual delivery effect of UAVs, the effective coverage rate *η*_1_, uncover rate *η*_2_, ineffective coverage rate *η*_3_, utilization rate of balls *η*_4_, and repetition rate of balls *η*_5_ are defined this article. Since the sum of *η*_1_ and *η*_2_ is 1, the changes of *η*_1_ and *η*_2_ in the exhibit an inverse synchronization relationship. So when quantifying the effectiveness of the operation, *η*_1_ is chosen as the system operational effect evaluation indicator; *η*_3_ reflects the degree of coverage of the balls beyond the boundary, even though there may be cases of balls coverage area exceeding the boundary, it will not cause drug damage like that caused by pesticide overreach spraying, so the impact of *η*_3_ is not considered in the evaluation process, the ineffective coverage situation is indirectly reflected by the utilization rate of balls *η*_4_, so *η*_4_ and *η*_5_ are used to evaluate the utilization effect of balls. The weight of each indicator is determined by the two-level indicator weighting method, and through linear weighting method, a comprehensive score is obtained to analyze the overall operational quality of the trichogramma balls UAV delivery system. this method provides a standard for a comprehensive and objective evaluation of the overall operational quality of the trichogramma balls UAV delivery system, and the defined indicators provide target parameters for the flight path planning of the trichogramma balls UAV delivery system.

The analysis of the system field operational effect reveals that a larger obtuse edge in the actual operational field boundary can result in a higher effective coverage area, at the right angle edge equal to 90°, it is easy to lead to less effective coverage area, and the more right angle edge, the less effective coverage area, and at the sharp angle edge less than 90°, the effective coverage area is increased when the ball drop point is pushed to the apex of the acute angle, because ineffective coverage of biological control does not lead to ecological deterioration, so the effect of the resulting high ineffective coverage is weakened in the comprehensive evaluation. Therefore, among the rectangular, trapezoidal and stepped fields, the trapezoidal fields operational effect is the best, followed by rectangular fields, and stepped fields operational effect is the worst. When comprehensively evaluating the effect of field operations and the utilization effect of balls, according to the scoring results, the comprehensive operational quality of the trapezoidal fields is the best, followed by the stepped, and the rectangular is the worst. In summary, it is better to divide the fields boundaries into trapezoids wherever possible when the UAV is operating in the area. This can achieve the best operating effect.

The employment of UAVs for the delivery of trichogramma balls to control insect pests has led to a reduction in pesticide usage, even in cases of redelivery or cross-border delivery, This method does not cause drug damage, thereby substantially enhancing the overall ecological benefits (Markle et al., 2016; Tang et al., 2018; Richardson et al., 2020; Fu et al., 2021; Li et al., 2021; Yan et al., 2021). The research conducted on this method and the evaluation indicators used to assess its operational effect have both provided technical support and a scientific basis for its application and promotion. This contributes to ecological protection in the agricultural field and the development of high-quality agriculture. Future work will focus on the path planning, flight route control, and factors affecting landing position during the UAV delivery process to achieve a higher operational quality and more precise pest control effects.

## Data availability statement

The original contributions presented in the study are included in the article/**Supplementary Material**. Further inquiries can be directed to the corresponding author.

## Author contributions

JLi contributed to conception and design of the study. ML and YQ organized the database. HX, ML, YQ, GF, YZ and JLv performed the statistical analysis. HX, ML, and YQ wrote the first draft of the manuscript. HX, ML, YQ, GF, YZ, and JLi wrote sections of the manuscript. All authors contributed to the article and approved the submitted version.
