# Supplementary material for: Design of a trichogramma balls UAV delivery system and quality analysis of delivery operation
Source: Front Plant Sci. 2023 Dec 5;14:1247169. doi: 10.3389/fpls.2023.1247169 (PMC10728877; doi:10.3389/fpls.2023.1247169)
Supplement: Supplementary file 1 [file Table_1.docx]

SUPPLEMENTARY TABLE 1 Technical Parameters of K705 Board.

| **Technical parameter** | **Parameter value** |
| --- | --- |
| Single point positioning accuracy | H<1.5 m, V<3 m (1δ, PDOP<4) |
| Static differential accuracy | H: ±(2.5+1×10^-6^×D) mm  V: ±(5.0+1×10^-6^×D) mm |
| RTK accuracy | H: ±(8.0+1×10^-6^×D) mm  V: ±(15+1×10^-6^×D) mm |
| Timing accuracy | 20 *ns* |

SUPPLEMENTARY TABLE 2 Number of released balls.

| **Steering engine**  **speed (r/min)** | **Number of balls released within 2 minutes** | | | | **Total number of balls released** | **Total number of blocked and missed balls** |
| --- | --- | --- | --- | --- | --- | --- |
|  | **Group 1** | **Group 2** | **Group 3** | **precision (%)** |  |  |
| 6 | 36 | 35 | 36 | 99.07% | 200 | 0 |
| 9 | 53 | 54 | 54 | 99.38% | 200 | 0 |
| 12 | 74 | 74 | 73 | 97.69% | 200 | 0 |
| 15 | 91 | 92 | 92 | 98.15% | 200 | 0 |
| 18 | 108 | 109 | 108 | 99.69% | 200 | 0 |

SUPPLEMENTARY TABLE 3 Experimental and analytical data on the spacing of trichogramma balls placement.

| Flight speed (m/s) | 3 | | 5 | | 7 | | 9 | |
| --- | --- | --- | --- | --- | --- | --- | --- | --- |
|  | Route 1 | Route 2 | Route 1 | Route 2 | Route 1 | Route 2 | Route 1 | Route 2 |
| Spacing 1(m) | 10.0 | 9.7 | 8.0 | 11.0 | 9.4 | 9.3 | 9.1 | 9.9 |
| Spacing 2(m) | 10.5 | 10.0 | 12.5 | 11.4 | 9.3 | 10.0 | 10.6 | 11.2 |
| Spacing 3(m) | 10.0 | 9.4 | 10.1 | 10.3 | 11.6 | 10.5 | 9.9 | 9.9 |
| Spacing 4(m) | 9.8 | 10.2 | 10.6 | 10.4 | 9.3 | 11.1 | 11.8 | 10.9 |
| Spacing 5(m) | 11.3 | 9.6 | 11.0 | 10.7 | 11.4 | 11.0 | 10.8 | 10.1 |
| Spacing 6(m) | 9.9 | 10.7 | 9.9 | 8.4 | 10.2 | 11.0 | 9.6 | 9.7 |
| Average spacing(m) | 10.3 | 10.0 | 10.4 | 10.4 | 10.2 | 10.5 | 10.3 | 10.3 |
| Sum of squares | 0.543 | | | | | | | |
| Mean square | 0.181 | | | | | | | |
| F | 0.231 | | | | | | | |
| Sig. | 0.874 | | | | | | | |
